# Antibacterial activity of ultrathin platinum islands on flat gold against *Escherichia coli*

**DOI:** 10.1038/s41598-020-66504-w

**Published:** 2020-06-12

**Authors:** Masataka Hakamada, Susumu Sakakibara, Naoki Miyazawa, Soichiro Deguchi, Mamoru Mabuchi

**Affiliations:** Department of Energy Science and Technology, Graduate School of Energy Science, Kyoto University, Yoshidahonmachi, Sakyo, 606-8501 Kyoto Japan

**Keywords:** Biomedical materials, Synthesis and processing

## Abstract

Nanoporous Au exhibits high antibacterial activity (AA) without releasing reactive oxygen species or metal ions, instead its AA depends on the work function (WF) because cell walls are affected by peculiar electronic states at the surface. Based on this mechanism, a flat surface without nanostructure should show high AA if the WF of the surface is suitably tuned. To verify this, ultrathin Pt islands with high WF was fabricated on flat Au by underpotential deposition (UPD) of copper and subsequent redox replacement with Pt, and the AA of the Pt/Au substrate on *Escherichia coli* was evaluated. The Pt/Au substrate showed higher AA than Pt and Au surfaces, and a positive relationship between AA and WF was demonstrated. In addition, first principles calculations were performed to investigate the mechanism for the high WF of the Pt/Au substrate. The findings suggest that the high WF of the Pt/Au substrate is at least partly due to charge transfer from Au to Pt.

## Introduction

Nanomaterials such as nanoparticles exhibit certain interesting materials properties that are not shown by the related bulk materials. The high antibacterial activity (AA) of nanomaterials is one of their notable properties^1–6^, and is generally related to the release of diffusive antimicrobial species such as reactive oxygen species (ROS) and metal ions. However, some nanoparticles exhibit prominent AA without releasing harmful species^7–10^. High AA without the release of harmful species is a result of dysfunction of cytoplasmic proteins owing to the ready cellular uptake of nanoparticles. However, it has been shown that nanostructured surfaces are also highly bactericidal^11–13^, and cellular uptake of nanostructured surfaces does not occur because their macroscopic side lengths are generally in the of order of millimeters or larger, making them unable to pass through the cell wall. Therefore, the disorder of cell walls via direct contact with substrate is suggested to be responsible for AAs of nanostructured surfaces. It was reported that nanoporous Au (npAu), which is typical of nanostructured surfaces, showed high AA against *Escherichia coli* (*E. coli*) and *Staphylococcus epidermidis*^14^. The AA of npAu was not due to the release of harmful diffusive species^14^. An experimental and computational study^15^ suggested that the cell wall is negatively hyperpolarized by npAu, which leads to dysfunction of membrane proteins such as the potassium channel.

Electrons spill out on a metallic surface and an electric double layer is formed, resulting in a positive metallic surface^16,17^. It is therefore suggested that the nature of peculiar electronic states at the surface is responsible for the AA of npAu. The work function (WF) is related to the electric double layer at the surface^18,19^, therefore the AA of npAu has a positive correlation with the WF because the WF is increased by the enhanced hyperpolarization of cell walls^20^. According to this mechanism, a flat surface without nanostructures should show high AA if the WF of the surface is suitably tuned. The scope of this work is to verify this hypothesis by investigating the AA of a flat noble metal surface with high WF.

One of the main methods of tuning the WF of a metal is adsorption of molecules. Computational studies have shown that a C_60_ monolayer on Ag (111) and Au (111) surfaces changed their WFs and the CO adsorption of the flat surfaces^21^; and that stepped and kinked surfaces reduced the WF of Au^22^. In addition, an experimental study revealed that adsorbed oxygen on Al (100) surfaces reduced the WF of Al^23^. It is therefore anticipated that the WF of noble metals can be tuned by stacking with non-metallic molecules. However, the effect of the non-metallic adsorbates themselves, which often and intrinsically have antibacterial capacity, on the AA cannot be neglected. Hence, the decorating element should be a noble metal with chemical inertness. Platinum (Pt) is suitable because the ion elution of Pt is strictly limited owing to its nobleness (or high standard electrode potential).

Underpotential deposition (UPD) is a phenomenon in which a metal ion in an aqueous solution is precipitated onto the surface of a dissimilar metal in a nobler potential region than an equilibrium potential for precipitation. Ideally, the deposited atoms form a monoatomic layer. Stacking by UPD is suitable for adjustment of the WF of a flat surface since UPD is related to the WF of surfaces^24,25^. In the present study, ultrathin Pt islands were dispersedly fabricated on flat Au substrate by UPD of copper and subsequent redox replacement with Pt, and the AA of the Pt/Au substrate against *E. coli* was compared with those of flat (bulky) Pt and Au substrates. In addition, their WFs were measured with photoemission yield spectroscopy, and the relationship between AA and WF was investigated. First principles calculations were also performed to reveal the mechanism for the high WF of the Pt/Au surface.

## Materials and Methods

### Experimental details

#### Preparation of Pt/Au, Au, and Pt surfaces

A radio-frequency sputtering apparatus was used to fabricate flat Au and Pt substrates. Au (>99.9 mass%) and Pt (>99.9 mass%) were sputtered onto a glass slide (2.5 × 2.5 mm and 1.2 mm thick), and flat Au and Pt surfaces with a thickness of 100 nm were fabricated. To fabricate a flat Pt/Au substrate, UPD and subsequent redox replacement were used^26^. First, UPD of copper was carried out using a potentiostat on flat Au. A typical three-electrode electrochemical cell with a platinum black counter electrode, a saturated calomel electrode (SCE) reference electrode, and the working electrode of flat Au (fabricated by the sputtering explained above) were used. Cyclic voltammetry (CV) was performed in a mixed aqueous solution of H_2_SO_4_ (0.5 M) and CuSO_4_ (5 mM) at 1 mV/s in a potential window of −0.2 to 0.6 V (vs. SCE) at room temperature. During CV, the peak for UPD of copper was observed in the current-potential curve^26^, as shown in Fig. S1. UPD of copper on the Au was achieved by stopping the CV at the end of this peak. Subsequently, the Au electrode modified by Cu UPD was rapidly transferred into a H_2_PtCl_6_ (1 mM) + HClO_4_ (0.1 M) solution and held for 10 min at room temperature for redox replacement of the Cu layer with Pt, to form a monolayer (ideally) of Pt on the Au surface. Thereafter, the Pt/Au substrate was washed ten times with distilled water to remove unreacted H_2_PtCl_6_ and HClO_4_.

#### Cyclic voltammetry (CV)

Electrochemical characterization of the surfaces was achieved by CV using a potentiostat. A typical three-electrode electrochemical cell with a platinum black counter electrode, a reference SCE, and Au or Pt/Au working electrode were used. CV was performed at 10 mV/s in a potential window of 0.0 to +1.6 V (vs. SCE) at room temperature.

#### Chemical composition analyses

The chemical compositions of the substrates were determined by X-ray photoemission spectroscopy (XPS, ULVAC-PHI, Chigasaki, Japan) under high vacuum.

#### Morphological analysis of Pt/Au surface

The surface morphology of the Pt/Au substrates was examined using a scanning probe microscope (SPM, SPM-9700, Shimadzu, Japan). Silicon nitride cantilevers with a nominal spring constant of 42 N m^−1^ were used. A phase detection mode where the cantilever oscillated during scanning was used.

#### Antibacterial activity (AA) tests

The antibacterial properties of the Pt/Au, Pt, and Au substrates were investigated according to the Japanese Industrial Standard (JIS) “Antibacterial products—Test for antibacterial activity and efficacy”^27^. First, one platinum loop of bacteria incubated in medium was removed from the colony and placed in 5 mL of 1/500 nutrient broth and then agitated using a vortex mixer. Second, 100 μL of the bacterial suspension was dropped onto the samples and then a 25 × 25 mm polyethylene film was used to cover the bacterial suspension. Bacterial suspensions were incubated on the specimens for 24 h in humidity-controlled incubators at 310 K and at a relative humidity of 50% to allow for contact of bacteria on the surface^14^. Third, the incubated bacteria were recovered using 10 mL of Soybean Casein Digest Broth with Lectithin & Polysorbate 80 (SCDLP) medium and diluted 10-fold in phosphate-buffered saline (PBS). The diluted PBS was mixed with LB medium to make a 10-fold dilution series of LB pour plates, which were then incubated at 310 K for 48 h. The number of colonies in the LB pour plates was then counted. Viable bacteria counts (VBCs) were statistically analyzed by one-way analysis of variance followed by a post-hoc test. When the Au surface was used as a standard, the AA of the Pt/Au and Pt surfaces was given by

1 $$AA={\rm{l}}{\rm{o}}{{\rm{g}}}_{10}({N}_{o}/N)$$

where *N*_0_ is the viable bacteria count for the Au surface and *N* is the viable bacteria count for the Pt/Au or Pt surfaces. The mean value of AA was obtained from 5 repeated tests. All results are expressed as mean ± standard deviation.

#### Photoemission yield spectroscopy in air (PYSA)

Photoemission yield spectra were obtained using a photoemission yield spectrometer (AC-2, Riken Keiki, Japan) to estimate the WF of the substrates. UV-rays emitted from a deuterium lamp were monochromated using a grating monochrometer and focused onto the surface via an optical fiber, where an energy increment of 0.05 eV for incident monochromated UV-rays was used. The number of photoelectrons emitted from the surface was counted using the open counter (LE-6100, Riken Keiki, Japan) in air. Photoemission yield measurements were made at room temperature. The threshold energy of photoemission, which corresponds to the work function, was determined from the energy of an intersecting point between a background line and the extended line of the square root of the photoemission yield.

#### Statistical analysis

The data are presented as means ± standard deviation. Statistical significance of viable bacterial counts was determined using Student’s *t*-test.

### Computational details

First principles calculations were performed for geometry optimization of the Au and Pt/Au surface models using the Cambridge Serial Total Energy Package (CASTEP)^28^, in which a plane-wave basis set was used to calculate the electronic properties based on density functional theory (DFT)^29^. The Perdew-Burke-Ernzerhof functional (PBE) version of the generalized gradient approximation^30^ was used to represent exchange and correlation interactions within the DFT. Ultrasoft pseudopotentials^31^ were used for all elements in the calculations. The cutoff energy was set to 320 eV and the Brillouin zone was sampled using 5 × 5 × 1 Monkhorst-Pack *k*-point meshes^32^. Periodic boundary conditions were applied in the *x*, *y*, and *z* directions for all of the calculations.

The Au surface model consisted of 4 atomic layers of 4 × 4 and a vacuum layer of 30 Å. The atoms in the top three layers were relaxed to their equilibrium positions and the atoms in the bottom layer were frozen at their bulk positions in the model. To create a Pt/Au surface model, a 2 × 2 Pt monolayer was placed on the top layer of the Au surface model. The Pt monolayer was positioned on four adsorption sites: fcc, hcp, top, and bridge sites (see Fig. S2(a–d)). The lowest internal energy was obtained at the fcc site (data not shown). Therefore, the model with a 2 × 2 Pt monolayer positioned on the fcc site was designated the Pt/Au model with low Pt coverage. The Pt/Au models with high and complete coverage of Pt were modeled by adding two and four Pt atoms to the Pt monolayer of the model with low Pt coverage, respectively, as shown in Fig. S3. Mulliken Population analysis was performed to investigate charge transfer between the Au and Pt atoms^33^.

## Results

### Surface characterization

**Figure 1:**
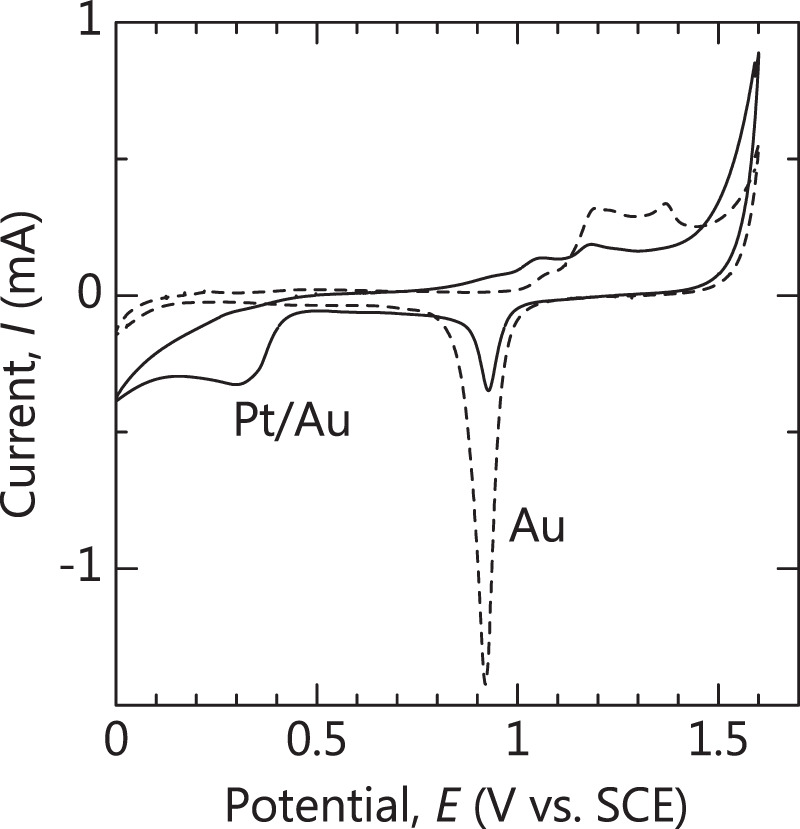
Cyclic voltammetry curves of Au and Pt/Au substrates.

The CV curves of the Au and Pt/Au substrates are shown in Fig. 1. The shape of the curve for the Au substrate is typical of a Au electrode. For the Pt/Au substrate, the reduction peak of Au at 0.92 V was attenuated and a new reduction peak centered at 0.30 V appeared. Considering the relationship between the RHE and SCE^34^, the peak at 0.30 V almost agrees with that of oxygen reduction on platinum^35^, indicating that the Cu on the Au surface was replaced with Pt. The fact that the peak for Au (around 0.90 V) was still detected after redox replacement suggests that the Au surface was not completely covered by a Pt layer.

**Figure 2:**
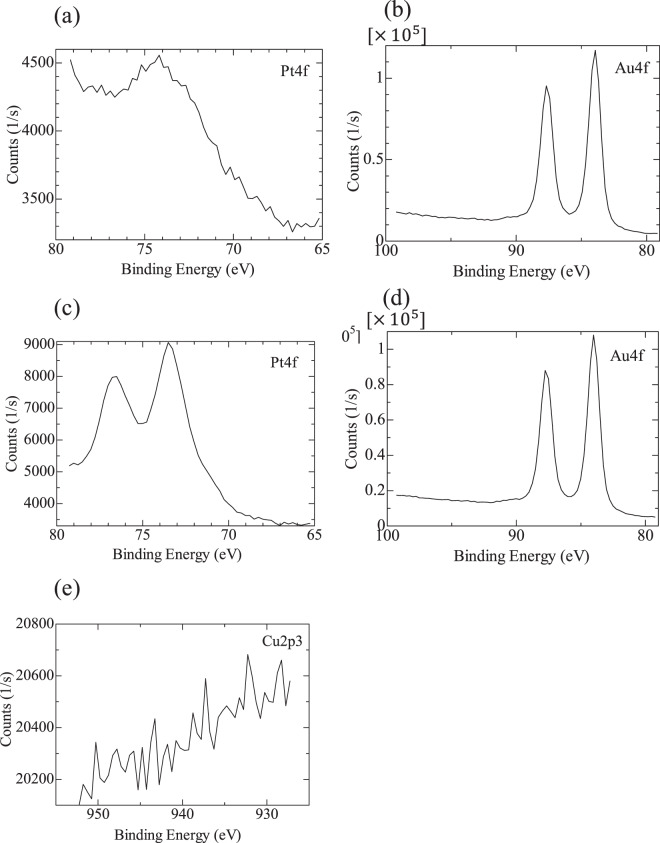
XPS binding energy spectra of (**a**) Pt4f for the Au substrate, (**b**) Au4f for the Au substrate, (**c**) Pt4f for the Pt/Au substrate, (**d**) Au4f for the Pt/Au substrate, and (**e**) Cu2p3 for the Pt/Au substrate.

XPS spectra of the Au and Pt/Au surfaces are shown in Fig. 2. A broad peak was found at 74 eV for the Au substrate, which corresponds to Au5p1/2; however, the Pt4f peak was not detected, as shown in Fig. 2(a). In contrast, the sharp Pt4f peak was detected for the Pt/Au substrate (Fig. 2(c)). In addition, the Cu2p3 peak was not observed for the Pt/Au substrate (Fig. 2(e)), showing that the Cu layer, which was precipitated by UPD was completely replaced with Pt. Au was detected for both the Au and the Pt/Au substrates (Fig. 2(b,d)). These findings suggest that platinum partially covered the surface of Au as a result of UPD and redox replacement, which agrees with the CV result.

**Figure 3:**
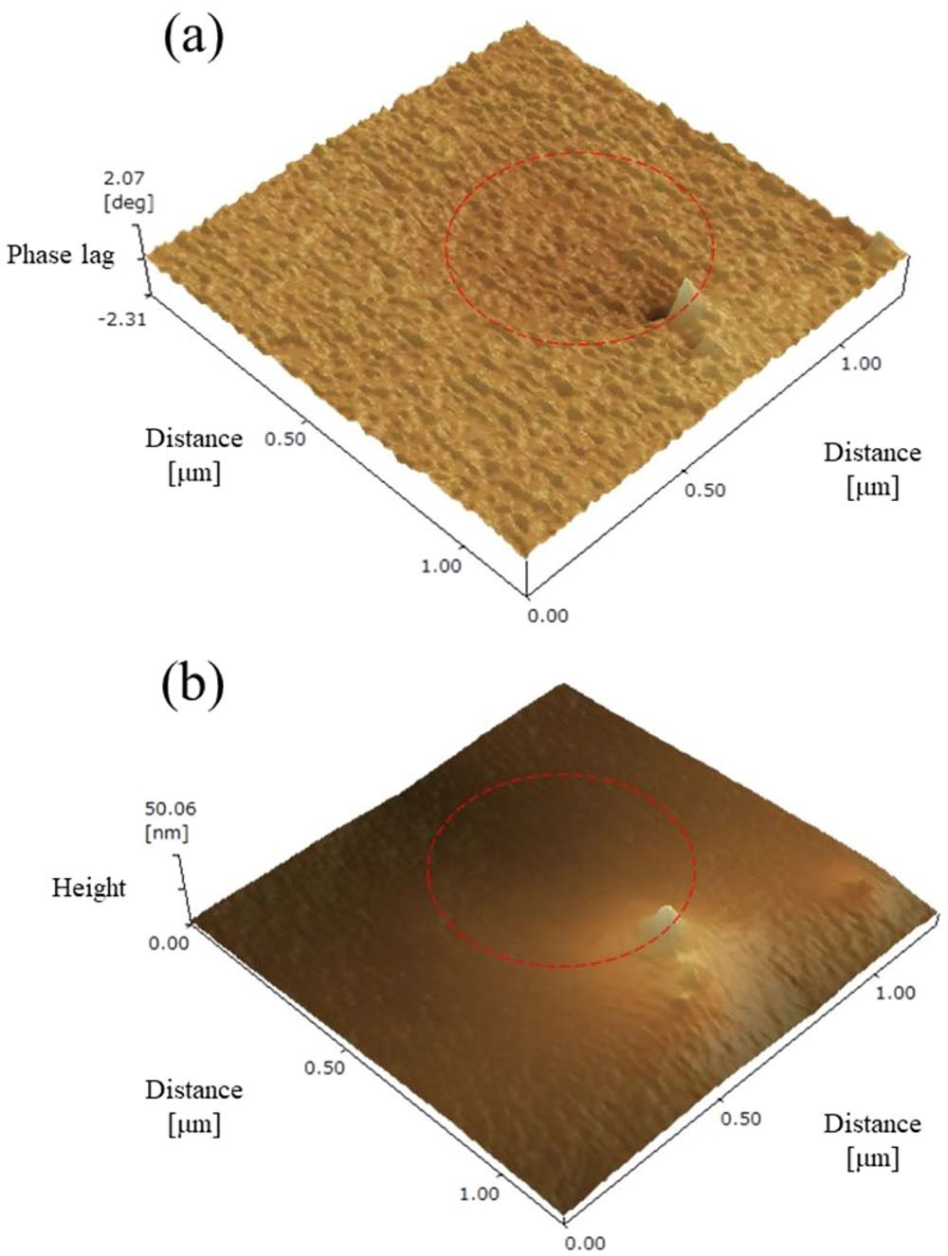
Surface morphology of the Pt/Au substrate by scanning probe microscopy, (**a**) phase image and (**b**) height image. An island of a phase that is different to the surrounding areas is indicated by a red circle.

Figure 3(a) shows an SPM phase image of the Pt/Au substrate. Islands of a phase that was different to the surrounding areas were observed. The difference in phase is related to the different physical properties of the islands and the surroundings. Figure 3(b) shows the height image of the Pt/Au substrate. Focusing on the island with different phase in Fig. 3(a), which is indicated by a red circle, a clear difference between the height of the islands and the substrate was not detected. Considering the precision of height detection in the SPM (=0.01 nm), this means that the deposited layer was very thin. Therefore, it is suggested that the thickness of the precipitated Pt layer was in the order of a monoatomic size, although the coverage was partial.

### Antibacterial properties

**Figure 4:**
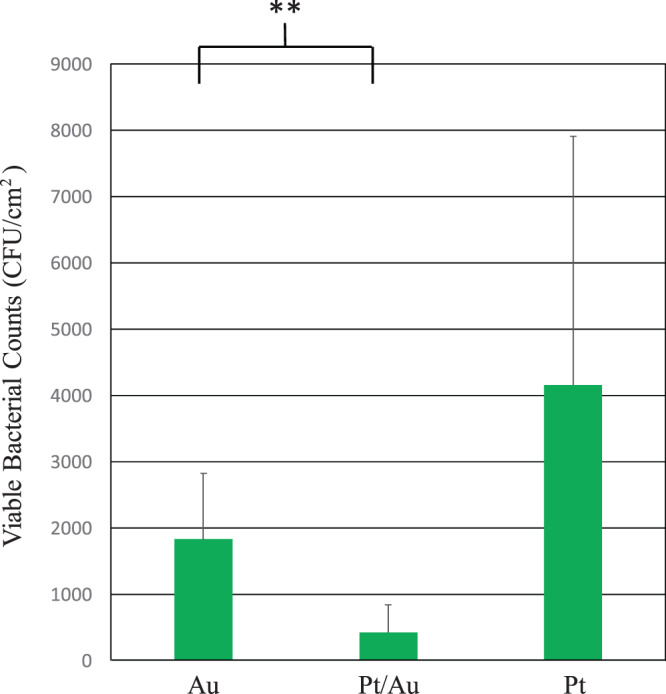
Viable bacterial counts of *E. coli* cultured for 24 h on Au, Pt/Au, and Pt substrates. **Indicates statistically significant differences for p-values < 0.1. The experiments were performed five times.

Figure 4 shows the viable bacterial counts (VBCs) of *E. coli* cultured on the substrates. It should be noted that the VBC of the Pt/Au substrate was lower than those of the Au and Pt substrates. Therefore, the monoatomic Pt lamination increased the AA of Au, indicating that nanostructure such as nanoporous structure is not necessarily required to obtain high AA.

### Relationship between antibacterial properties and work function

The WF was measured by the linear approximation of the background and (yield^0.5^)-increase region, based on the relationship between the square roots of the photoemission yield and energy of incident electrons by PYSA measurement (Fig. S4). As a result, the WFs were found to be 4.72 eV for the Au substrate, 4.78 eV for the Pt substrate, and 5.18 eV for the Pt/Au substrate. The WFs of the Au and Pt substrates agreed approximately with those in the previous work^36^. It should be noted that the WF of the Pt/Au substrate was larger than those of the Au and Pt substrates. When a thick layer is deposited on a substrate, the WF of the deposition/substrate will become the same as that of the deposition. The observation that the WF of the Pt/Au substrate was larger than that of the Pt substrate, suggests that the Pt layer of the Pt/Au substrate is very thin, which agrees with the result of the height image of the Pt/Au substrate (Fig. 3(b)).

**Figure 5:**
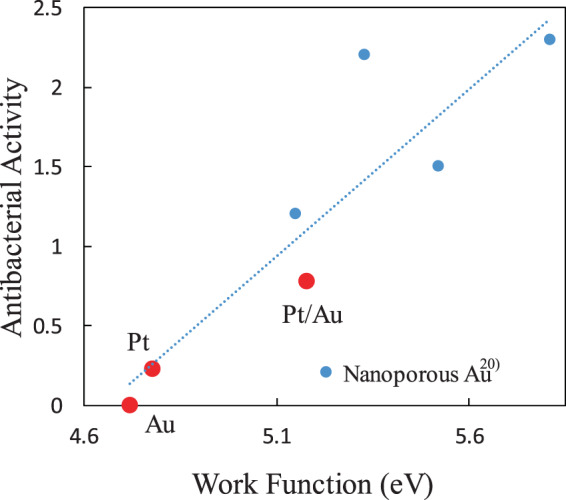
Relationship between antibacterial activity and work function for Pt/Au, Au, and Pt substrates. The data for nanoporous Au (ref. ^20^) are also shown.

Figure 5 shows the relationship between the WF and AA for the Au, Pt, and Pt/Au substrates. The data for npAu^20^ are also shown in Fig. 5 for comparison. There appears to be a positive correlation between the WFs and AAs, which also includes the data for npAu. An increase in the WF is related to an enhanced electric double layer at the substrate that leads to hyperpolarization of cell walls, which is responsible for cell death^15^. Therefore, it should again be noted that nanostructure, such as nanoporous structure, is not necessarily needed to obtain high AA, and that tuning the electronic state at the surface is in fact the critical factor.

## Discussion

As shown above, the AA depended on the tuning of the WF, which is related to the electronic state at the surface. It is therefore important to investigate the electronic state of the Pt/Au substrate. In the present study, first principles calculations were performed to investigate the electronic state of the Pt/Au substrate.

It has been reported that large lattice disorders were generated at the surface of nanoporous metals^37,38^. Compressive disorder increases the WF^39,40^, therefore if compressive disorders are generated at the Pt/Au substrate, the WF should be large. Because the atomic size of Au is larger than that of Pt, the compressive lattice disorder tends to be generated in the Au of the Pt/Au substrate. However, the tensile lattice disorder should be generated in the Pt of the Pt/Au substrate. Some bacteria adhere to the Pt of the Pt/Au substrate, therefore the high AA of the Pt/Au substrate cannot be fully explained by the surface disorders.

**Figure 6:**
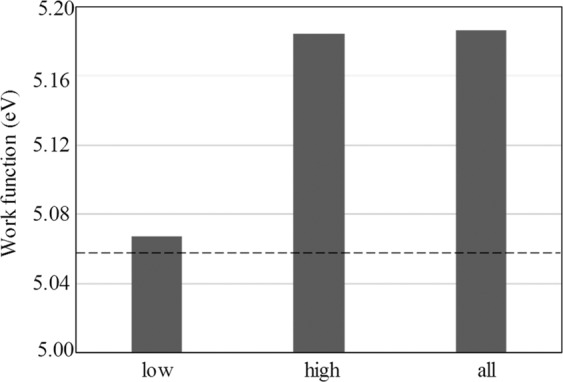
Work functions by first principles calculations for three Pt/Au models, where “low” is the Pt/Au model with low Pt coverage, “high” is the Pt/Au model with high Pt coverage, and “all” is the Pt/Au model with complete Pt coverage. The dashed line indicates the work function of the Au surface without Pt.

Generally, the WF can be given by^40^

2 $$\phi =-\mu +D$$

where $\phi$ is the WF, $\mu$ is the component related to the intrinsic bulk electronic structure, and *D* is the component related to the dipole barrier on the structure. The second term (*D*) can give a stronger contribution to the WF compared with the first term ($\mu$). The second term depends on the charge density^40^, therefore the charge transfer between Pt and Au in the Pt/Au surface can affect the WF of the surface. To investigate this, first principles calculations were performed on the WF of three Pt/Au models (Fig. S3). In the calculations, the volume of the cell model was fixed: the lattice disorders were very low and the effect of disorder on the WF was not considered. The WFs calculated by the first principles calculations are shown in Fig. 6. The WFs of the three Pt/Au models were all larger than that of Au, which agrees with the experimental results. This suggests that the larger WF of the Pt/Au surface can be explained without consideration of the surface disorder. It can be seen from Fig. 6 that although the WF increases with the coverage of Pt, the WF is saturated at a certain Pt coverage

The electron charge transfer between Au and Pt in the three Pt/Au models was investigated by the Mulliken Population analysis. The results are shown in Tables 1–3, where the number of each atom is shown in Figs. S5 and S6. Interestingly, charge transfer from Au to Pt occurred in many atoms of the Pt/Au models, although charge transfer from Pt to Au was also found for some atoms—particularly in the Pt/Au model with low Pt coverage. The charge transfer from Au to Pt cannot be explained on the basis of electronegativity because the electronegativity of Au is larger than that of Pt. The WF increases with the charge density^40^, therefore the charge transfer from Au to Pt can lead to an increase in the WF of Pt in the Pt/Au surface. The trend of charge transfer from Au to Pt was enhanced in Pt/Au models with high Pt coverage ratio, which corresponds to the larger WF of the models with high Pt coverage ratio.

**Table 1:** Variations in charge of each atom in a Pt/Au model with low Pt coverage, where the number of each atom is shown in Fig. S5. The positive/negative variation indicates that the charge is decreased/increased, respectively.

Atoms	Charge variation
Pt1	0.00
Pt2	0.01
Pt3	0.02
Pt4	−0.01
Au28	−0.09
Au29	−0.05
Au30	0.03
Au31	−0.05
Au32	0.03
Au33	−0.07
Au34	−0.15
Au35	−0.06
Au36	−0.11

**Table 2:** Variations in charge of each atom in a Pt/Au model with high Pt coverage, where the number of each atom is shown in Fig. S6. The positive/negative variation indicates that the charge is decreased/increased, respectively.

Atoms	Charge variation
Pt1	−0.03
Pt2	−0.07
Pt3	−0.03
Pt4	−0.03
Pt5	−0.07
Pt6	−0.07
Au28	−0.08
Au29	0.03
Au30	0.07
Au31	0.03
Au32	0.13
Au33	−0.07
Au34	−0.03
Au35	−0.07
Au36	0.03

**Table 3:** Variations in charge of each atom in a Pt/Au model with complete Pt coverage. The positive/negative variation indicates that the charge is decreased/increased, respectively.

Atoms	Charge variation
Pt	−0.11
Au	0.12

**Figure 7:**
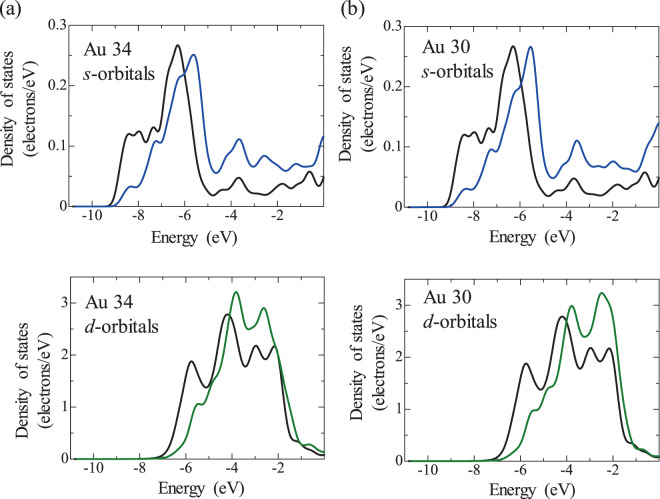
Density of states (DOS) of Au atoms in the Pt/Au model with low Pt coverage, (**a**) Au34 and (**b**) Au30, where the blue and green curves indicate the *s* and *d* orbitals, respectively. For comparison, the DOS of a Au atom that is not affected by Pt is included (black curves). The location of Au34 and Au30 is shown in Fig. S5. Au34 is the Au atom where charge is transferred from Pt to Au and Au30 is the Au atom where charge is transferred from Au to Pt.

**Figure 8:**
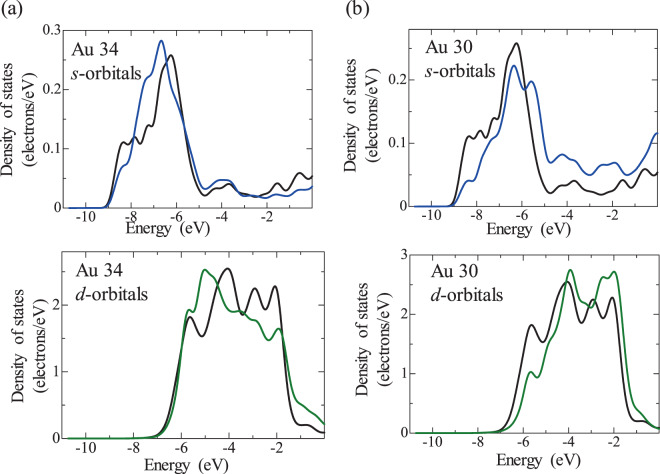
Density of states (DOS) of Au atoms in the Pt/Au model with high Pt coverage, (**a**) Au34 and (**b**) Au30, where the blue and green curves indicate the *s* and *d* orbitals, respectively. For comparison, the DOS of a Au atom that is not affected by Pt is included (black curves). The location of Au34 and Au30 is shown in Fig. S6. Au34 is the Au atom where charge is transferred from Pt to Au and Au30 is the Au atom where charge is transferred from Au to Pt.

**Figure 9:**
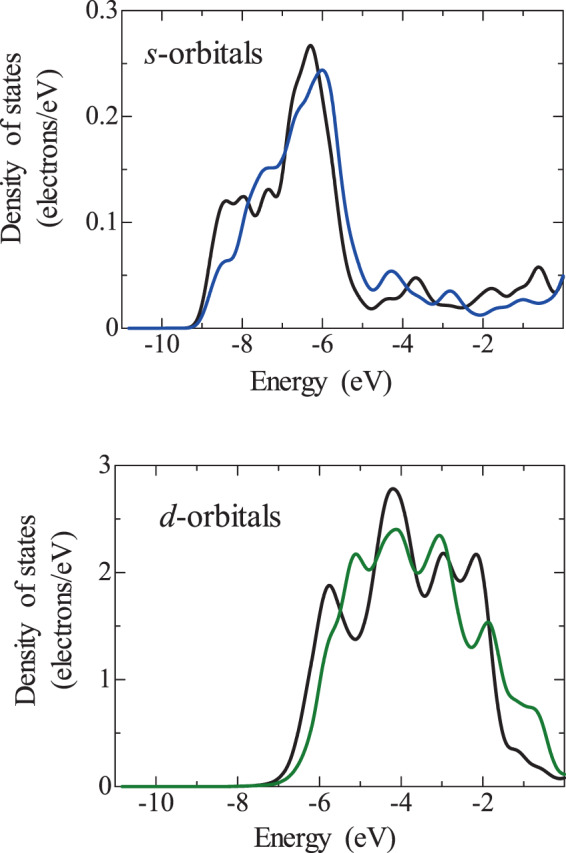
Density of states (DOS) of Au atoms in the Pt/Au model with complete Pt coverage, where the blue and green curves indicate the *s* and *d* orbitals, respectively. For comparison, the DOS of a Au atom that is not affected by Pt is included (black curves).

The DOS of Au atoms in the Pt/Au models are shown in Figs. 7–9, where Au34 is the Au atom where charge is transferred from Pt to Au and Au30 is the Au atom where charge is transferred from Au to Pt. The energy level of the electrons tended to shift to higher energies in the case of charge transfer from Au to Pt. It is therefore suggested that the charge transfer from Au to Pt occurs because the electrons of Au become unstable as a result of binding with Pt.

## Conclusions

The AA of a Pt/Au substrate having ultrathin Pt islands on flat Au with high WF was investigated to verify that flat substrates without nanostructure can exhibit high AA if the WF of the surface is suitably tuned. The flat Pt/Au substrate was fabricated by UPD of copper and subsequent redox replacement with Pt. Pt layers on the Au surface were very thin, although coverage was partial.

Viable bacteria count tests showed that the Pt/Au substrate exhibited higher AA than Pt and Au substrates. As a result, a positive relationship between AA and WF was found, the nanoporous structure is not necessarily needed to obtain high AA.

First principles calculations suggested that the high WF of the Pt/Au substrate is partly due to charge transfer from Au to Pt. The charge transfer from Au to Pt is interesting because it cannot be explained on the basis of electronegativity.

## Supplementary information

Supplementary Information.
