# Supplementary material for: Antibacterial activity of ultrathin platinum islands on flat gold against Escherichia coli
Source: Sci Rep. 2020 Jun 12;10:9594. doi: 10.1038/s41598-020-66504-w (PMC7293303; doi:10.1038/s41598-020-66504-w)
Supplement: Supplementary file 1 — Supplementary Information. [file 41598_2020_66504_MOESM1_ESM.pdf]

Supplementary Information for: Antibacterial activity of ultrathin platinum islands on flat gold against *Escherichia coli*

Masataka Hakamada, Susumu Sakakibara, Naoki Miyazawa, Soichiro Deguchi and Mamoru Mabuchi*

Department of Energy Science and Technology, Graduate School of Energy Science, Kyoto University, Yoshidahonmachi, Sakyo, 606-8501 Kyoto, Japan

* Corresponding author. Tel: +81-75-753-5606; e-mail: hakamada.masataka.3x@kyoto-u.ac.jp (M. Hakamada).

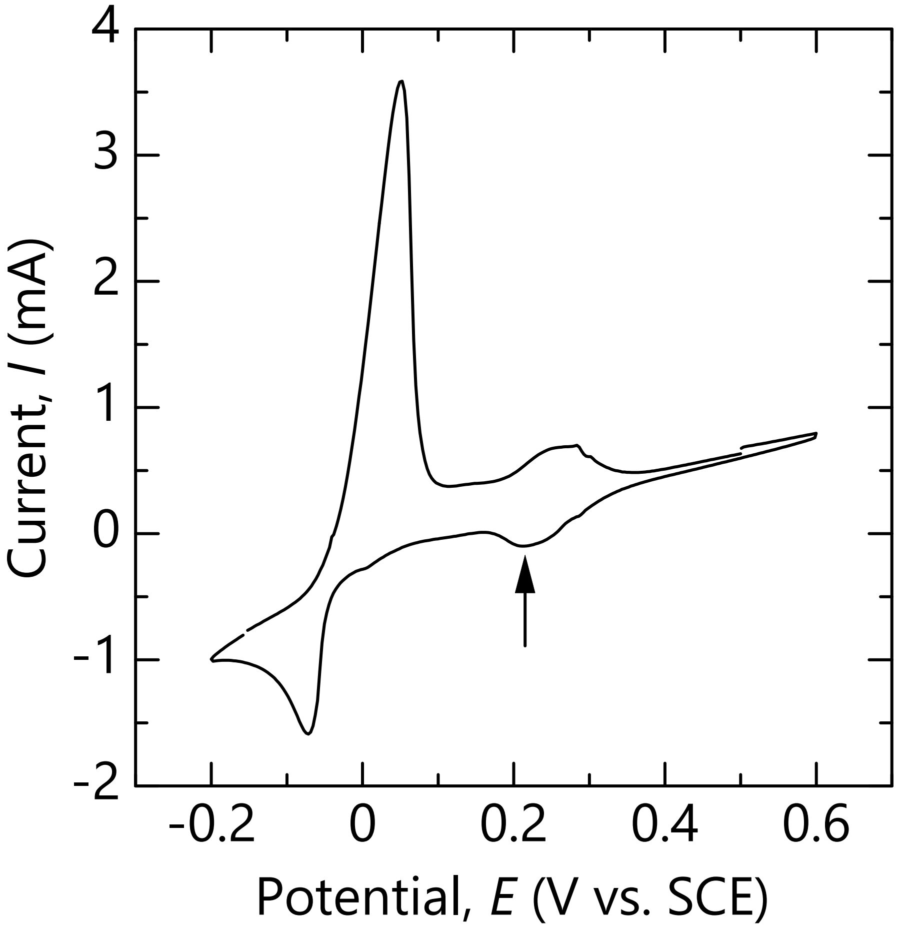

Figure S1 Cyclic voltammetry curve for underpotential deposition (UPD) of copper. An arrow indicates the peak for UPD of copper.

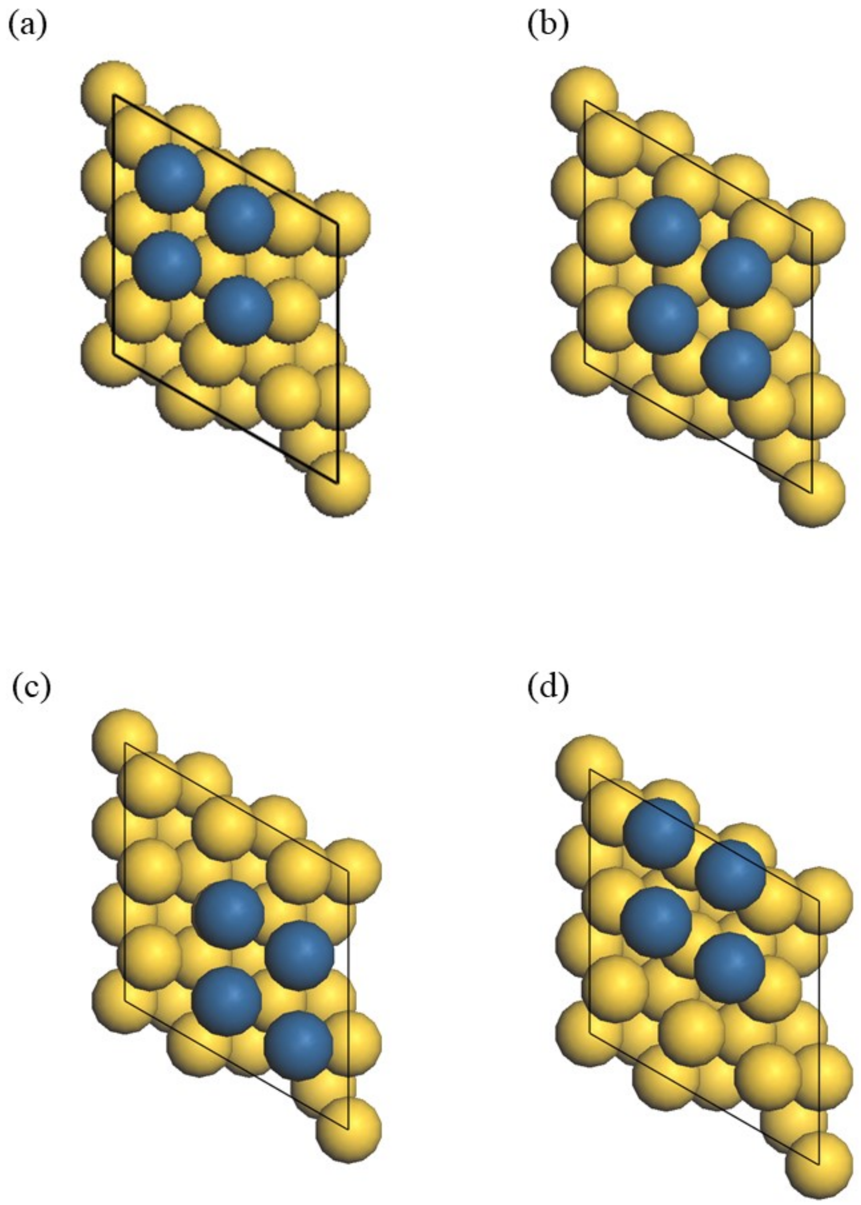

Figure S2 Pt/Au models with 2×2 Pt monolayer used for determination of adsorption site, (a) fcc site, (b) hcp site, (c) top site, and (d) bridge site. The yellow and blue spheres indicate Au and Pt atoms, respectively.

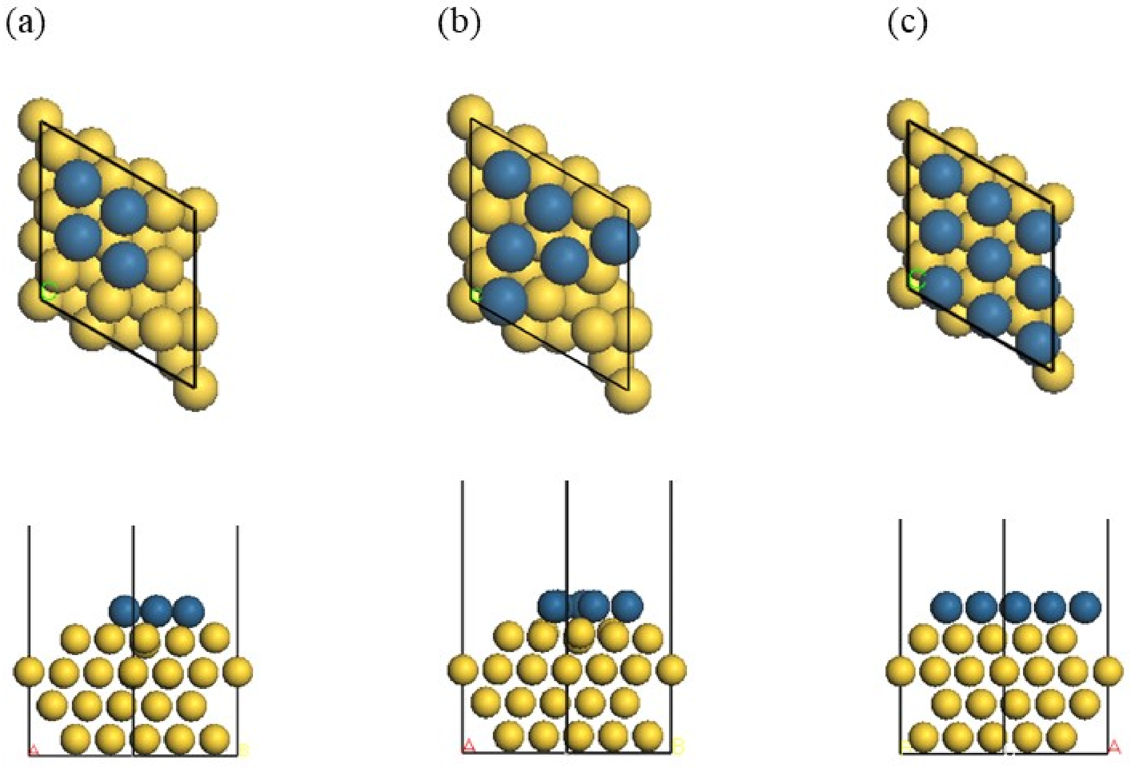

Figure S3 Pt/Au models for first principles calculations, (a) the model with low Pt coverage, (b) the model with high Pt coverage, and (c) the model with complete Pt coverage. The yellow and blue spheres indicate Au and Pt atoms, respectively.

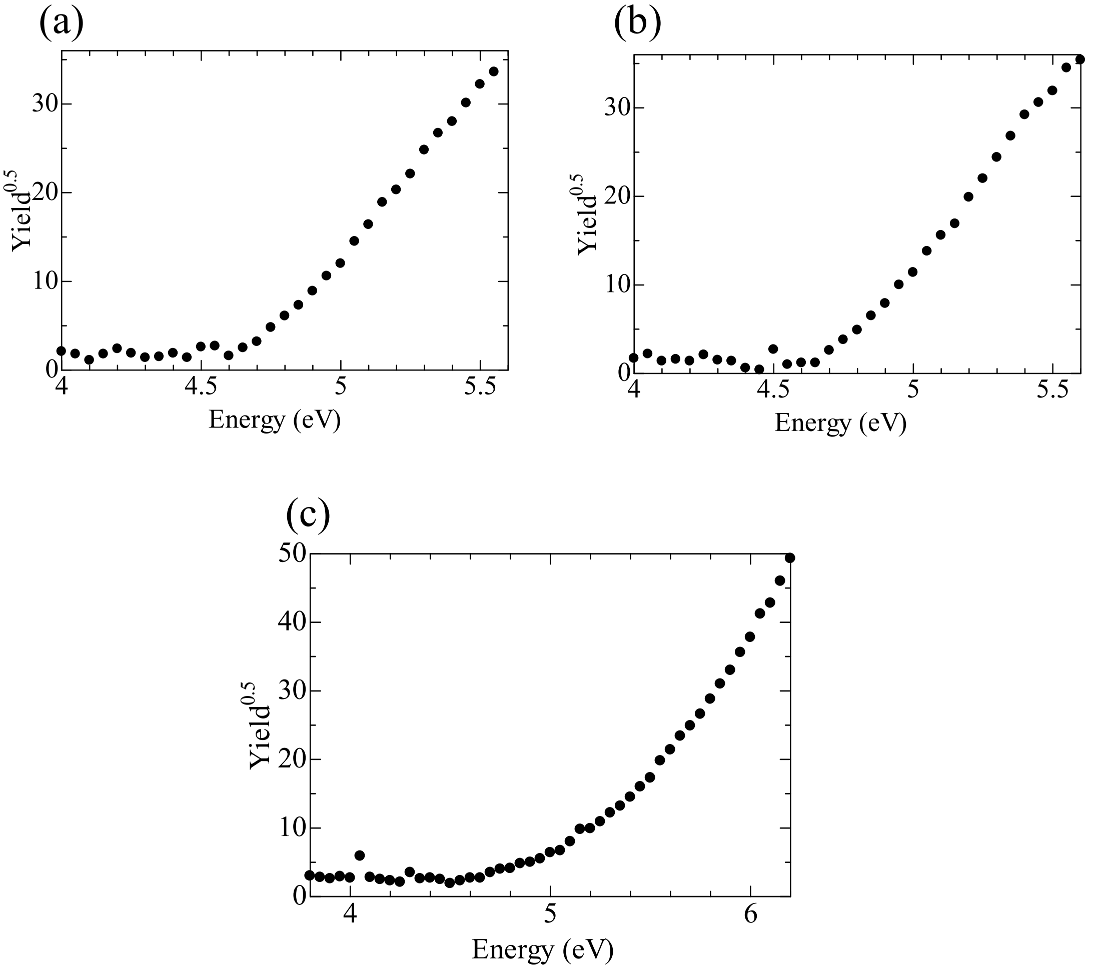

Figure S4 Photoemission yield of (a) the Au surface, (b) the Pt surface, and (c) the Pt/Au surface.

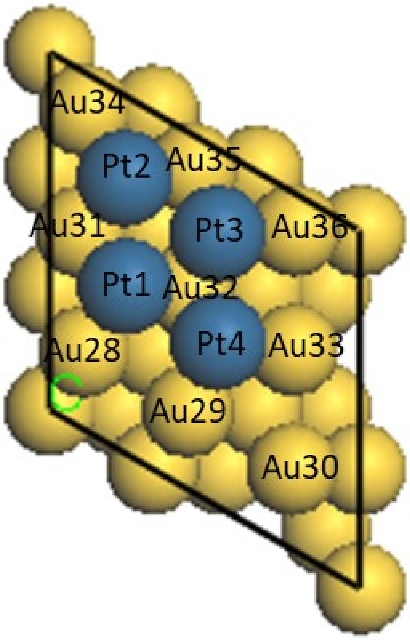

Figure S5 The number of each atom for Mulliken Population analysis in the Pt/Au model with low Pt coverage. The yellow and blue spheres indicate Au and Pt atoms, respectively.

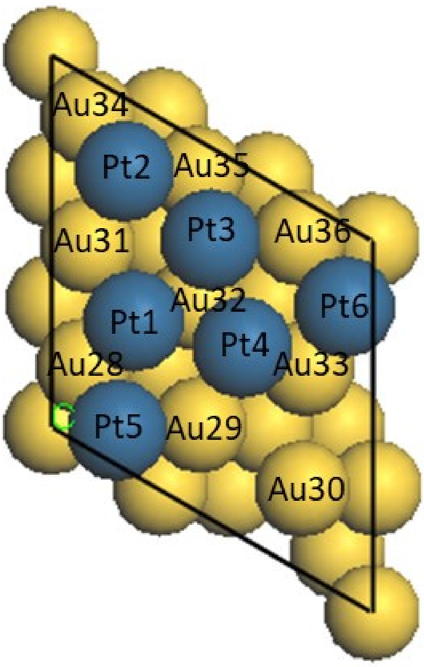

Figure S6 The number of each atom for Mulliken Population analysis in the Pt/Au model with high Pt coverage. The yellow and blue spheres indicate Au and Pt atoms, respectively.
